# Epidemiology and Genetic Epidemiology of the Liver Function Test Proteins

**DOI:** 10.1371/journal.pone.0004435

**Published:** 2009-02-11

**Authors:** Nilufer Rahmioglu, Toby Andrew, Lynn Cherkas, Gabriela Surdulescu, Ramasamyiyer Swaminathan, Tim Spector, Kourosh R. Ahmadi

**Affiliations:** 1 Department of Twin Research and Genetic Epidemiology, King's College London, London, United Kingdom; 2 Chemical Pathology, Guy's and St Thomas' Hospitals Trust, London, United Kingdom; Institute of Preventive Medicine, Denmark

## Abstract

**Background:**

The liver function test (LFT) is among the most commonly used clinical investigations to assess hepatic function, severity of liver diseases and the effect of therapies, as well as to detect drug-induced liver injury (DILI).

**Aims:**

To determine the relative contribution of genetic and environmental factors as well as test and quantify the effects of sex, age, BMI and alcohol consumption to variation in liver function test proteins - including alanine amino transaminase (ALT), Albumin, gamma glutamyl transpeptidase (GGT), total bilirubin, total protein, total globulin, aspartate transaminase (AST), and alkaline phosphotase (ALP) - using the classical twin model.

**Methods:**

Blood samples were collected from a total of 5380 twin pairs from the TwinsUK registry. We measured the expression levels of major proteins associated with the LFT, calculated BMI from measured weight and height and questionnaires were completed for alcohol consumption by the twins. The relative contribution of genetic and environmental factors to variation in the LFT proteins was assessed and quantified using a variance components model fitting approach.

**Results:**

Our results show that (1) variation in all the LFTs has a significant heritable basis (h^2^ ranging from 20% to 77%); (2) other than GGT, the LFTs are all affected to some extent by common environmental factors (c^2^ ranging from 24% to 54%); and (3) a small but significant proportion of the variation in the LFTs was due to confounding effects of age, sex, BMI, and alcohol use.

**Conclusions:**

Variation in the LFT proteins is under significant genetic and common environmental control although sex, alcohol use, age and BMI also contribute significantly to inter-individual variation in the LFT proteins. Understanding the underlying genetic contribution of liver function tests may help the interpretation of their results and explain wide variation among individuals.

## Introduction

The liver function test (LFT) is a routinely ordered clinical investigation used to assess hepatic function, disease severity [1]–[5], and to evaluate response to treatment by measuring the levels of various biomarkers (proteins) in the blood. These proteins reflect different aspects of a normal functioning liver [6]–[8]. For example, normal bilirubin (BILIRB) levels reflect adequate excretion of anions, normal alanine amino transaminase (ALT) or aspartate transaminase (AST) levels indicate hepatocellular integrity, and BILIRB or alkaline phosphotase (ALP) levels provide insights into adequate formation and flow of bile and albumin for protein synthesis.

In the clinic, LFT results are compared to and interpreted according to reference charts containing internationally accepted normal-levels for these proteins. However many patients are investigated for having levels outside the normal range without any obvious pathology or responsible factor being detected. The most common biomarkers that are routinely measured as part of the LFT include proteins which are specifically produced by the liver – such as ALT and albumin (ALB) – as well as those that are more ubiquitously produced, for example AST, which is also produced by red blood cells and cardiac/ skeletal muscles, and ALP which is also produced in bones and kidneys.

At the population level, previous studies have shown that some of the LFT proteins are highly variable, affected by various epidemiological factors including body mass index (BMI) [9], [10], alcohol consumption [11], [12], and age [10], [13], [14], and that at least some of these proteins are also influenced to some degrees by genetic factors [11], [15].

Twin studies provide one of the best epidemiological designs to gain insights into the contribution of genetic and environmental factors to population variation in clinical or evolutionary traits. Here, we use the classical twin design to investigate the relative contributions of genetic and environmental factors to variation in the most common liver biomarkers including alanine amino transaminase (ALT), albumin (ALB), gamma glutamyl transpeptidase (GGT), total bilirubin (TBILIRB), total protein (TPROT), total globulin (TGLOB), aspartate transaminase (AST) and alkaline phosphotase (ALP). Furthermore, we directly test for the effect of age, sex, BMI, and alcohol use on variation in the LFT proteins.

## Materials and Methods

### Ethics Statement

The study was approved by St. Thomas' Hospital Research Ethics Committee (EC96/439 Twins UK) and all participants provided written informed consent.

### Subjects and Questionnaire

A total of 5380 twins, comprising of 1804 DZ pairs and 886 MZ pairs (female to male ratio of approximately 10∶1), from the TwinsUK database at St. Thomas' Hospital [16] with mean age of 47 (range, 18–81years) were included in our study. Fasting blood samples were collected from all twins for biochemical assays of the liver function proteins and weight and height were measured at the visit (BMI was calculated as weight (kg)/height (m^2^)). Summary characteristics of the cohort and the measured proteins are presented in Table 1. Alcohol consumption data was collected from self-reported questionnaires. Questionnaire frequencies of recalled intake of beer, wine, fortified wine and spirits were converted into units of alcohol per week (with one unit equal to half a pint of beer, one glass of wine, or one measure of spirits) [17].

**Table 1:** Descriptive statistics of the raw data for female and male MZ and DZ twins including total number of individuals (n), means and standard deviations (s.d.) for the liver function test proteins and the major covariates included in the study.

Protein	Female				Male			
	MZ		DZ		MZ		DZ	
	n	Mean (s.d.)	n	Mean (s.d.)	n	Mean (s.d.)	n	Mean (s.d.)
**Age (18–81 yrs)**	1660	48.54 (13.57)	3320	46.52 (11.88)	112	43.76 (15.90)	288	48.34 (13.38)
**BMI**	1291	24.69 (4.50)	3091	25.21 (4.69)	95	26.27 (4.11)	278	26.24 (3.59)
**Alcohol Use**	1331	5.37(7.40)	2553	5.37(7.14)	58	11.51(10.91)	141	12.46(15.86)
**ALT**	1654	24.45 (12.19)	3302	25.45 (12.97)	112	27.49 (21.62)	285	34.65 (16.71)
**ALB**	1652	42.31 (3.23)	3302	41.82 (3.04)	110	45.25 (3.27)	287	42.78 (3.01)
**GGT**	1081	25.35 (22.05)	3032	25.35 (21.96)	63	32.66 (16.46)	274	44.62 (39.13)
**BILIRB**	1492	8.58 (3.61)	3282	8.70 (3.65)	63	12.90 (4.83)	274	12.41 (4.88)
**TPROT**	1490	71.26 (4.64)	3258	70.86 (5.19)	63	72.27 (3.42)	275	71.34 (4.89)
**TGLOB**	1486	29.41 (3.43)	3242	29.08 (3.88)	63	28.65 (3.28)	275	28.76 (4.19)
**AST**	98	10.31 (4.60)	477	9.74 (4.18)	-	-	-	-
**ALP**	1497	67.58 (19.84)	3285	67.08 (20.30)	63	72.99 (17.62)	275	74.66 (18.02)

Note AST was not measured in male twins.

### Biochemical Assays

All assays were performed on a Synchron LX20 automated multi channel analyzer (Beckman Coulter, Fulleton, CA). ALT activity was measured by a kinetic rate method (within run precision 4.3 and between run 4.6 at 20 iu/L). Serum albumin concentration was measured by means of a bichromatic digital endpoint methodology using bromcresol purple (BCP) reagent (within run precision 0.2 and between run 1.3 at 48 g/L). GGT activity was measured by an enzymatic rate method (within run precision 3.5 and between run 5.3 at 86 iu/L). BILIRB was measured by a timed endpoint Diazo method (within run precision 3.0 and between run 4.5). TPROT concentration was measured by means of a rate biuret method (within run precision 0.7 and between run 0.87 at 75 g/L). AST activity was measured by an enzymatic rate method (within run precision 0.7 and between run 1.0 at 175 iu/L). ALP activity was measured by a kinetic rate method using a 2-amino-2-methyl-1-propanol (AMP) buffer (within run precision 1.8 and between run 2.1 at 150 iu/L).

### Twin Studies

Twin studies are the optimum epidemiological design to study and partition population variation of a trait into genetic and non-genetic - shared and unique environmental - components. The classical twin design assumes that monozygotic twins share 100% of their genes and shared environment whereas dizygotic twins share on average 50% of their genes and 100% of shared environment. Thus any greater similarity between MZ as compared to DZ twin pairs is attributed to genetic factors [18], [19].

### Statistical Analysis

All measures of liver function test proteins were recorded as continuous variables. However, many were not normally distributed so appropriate statistical transformations were carried out prior to analyses; Total protein, GGT, TGLOB and ALP were log_10_ transformed, ALT, BILIRB, TPROT and AST were square root transformed, and ALB was normally distributed. Covariates age, BMI and alcohol consumption were all continuous variables as well.

Demographic differences between MZ and DZ twins were assessed by comparing means, medians and standard deviations (Table 1). We carried out logistic regression analyses - treating sex and zygosity as the explanatory variables - to check if age, BMI, and alcohol consumption as well as the levels of the LFT proteins were significantly different between male/female twins and MZ/DZ twins.

Ordinary least squares regression was used for analysis of covariates, age, BMI and sex. For alcohol consumption, truncated Gaussian regression was used to assess the independent contribution of alcohol consumption to variation of protein assay. Relatedness between the twins was accounted for using the “cluster” option in STATA. All preliminary analyses were performed using STATA 10 [20].

### Path analysis and genetic model fitting of twin data

Standard methods of quantitative genetic analysis were used to model latent genetic and environmental factors influencing sibling covariance for MZ and DZ twins. Genetic model fitting (path analysis) was used for the decomposition of the observed phenotypic variance (P) into additive (A) & dominant (D) genetic components and environmental components shared by both twins (C) & unique to each twin (E). The latter also includes measurement error. Dividing each of these components by the total variance yields the different standardized components of variance. For example the (narrow sense) heritability (h^2^) can be defined as the proportion of the total variance attributable to additive genetic variation or h^2^ = A/P. The significance of the variance components A, C, and D are assessed by comparing the model fit between a full model and a nested model in which each term is sequentially set to zero. Model fit is assessed using the likelihood ratio test. The purpose of the model fitting procedure is to explain the pattern of observed variances and covariances as accurately as possible. Genetic modeling was carried out using the computer package Mx, which has been specifically designed for analysis of twin and family data [21].

The effects of significantly associated covariates on LFT proteins, particularly age and BMI were removed by regressing LFT phenotype upon the covariates and model fitting was then performed using the residuals.

## Results

The age range of twins in the study was 18–81 years. Table 1 summarizes the characteristics of this cohort including total number of individuals (n), the mean (±s.d.) values of the LFT proteins for MZ/DZ, male/female twins.

The mean and standard deviations of the LFT proteins, BMI, alcohol consumption, and age range for the MZ/DZ twin populations within each sex group were comparable (p>0.05). Significant differences by zygosity were noted for ALT in the male twin cohort (p<10^−4^), although explicitly modeling the different MZ/DZ means and variances did not qualitatively affect the results in this case (data not shown).

### Sex effects on the LFT enzymes

Age was comparable between male and female twin pairs (χ^2^ = 0.04, p<0.83). As expected we observed a significant difference in BMI (χ^2^ = 21.29, p<10^−5^) and alcohol consumption (χ^2^ = 69.39, p<10^−4^) between the male and female twins. Overall, male twins had a higher BMI and consumed significantly more alcohol than female twins.

Except for TPROT and TGLOB, significant differences between the sex groups was observed for all other LFT proteins (p<1×10^−4^) with the proportion of variability explained (R^2^) ranging from 1% in the case of ALP to 6% for GGT and BILIR (Table 2); Note that AST was not measured in males.

**Table 2:** Results of the logistic regression analyses of sex on liver function test proteins.

	Sex			
	N	F	p	R^2^
**ALT**	5353	40.30	1×10^−4^	0.02
**ALB**	5351	46.22	1×10^−4^	0.02
**GGT**	4450	152.5	1×10^−4^	0.05
**BILIRB**	5111	158.8	1×10^−4^	0.06
**TPROT**	5086	2.73	0.1	–
**TGLOB**	5066	2.94	0.1	–
**AST**	–	–	–	–
**ALP**	5120	59.47	1×10^−4^	0.01

### Relationship between age, BMI, alcohol consumption, and the LFT enzymes

As the mean and range of age was comparable across the male/female sex strata we report results from pooled analyses (Table 3). Age was shown to have a small but significant effect on all the LFT enzymes except ALB and AST in the full cohort (p<0.005, R^2^ ranging from less than 1% to up to 3% in the case of ALP).

**Table 3:** Results of the linear regression analyses of age, BMI and alcohol consumption on liver function test proteins.

	Age				Body Mass Index (BMI)								Alcohol consumption†					
	Pooled				Females				Males				Females			Males		
	N	F¶	p	R^2^	N	F	p	R^2^	N	F	p	R^2^	N	Chi^2^	p	N	Chi^2^	p
**ALT**	5353	12.41	4×10^−4^	<0.01	4364	68.32	1×10^−4^	0.02	371	30.50	1×10^−4^	0.11	3864	0.15	0.70	197	0.62	0.43
**ALB**	5351	1.88	0.17	–	4363	17.71	1×10^−4^	<0.01	373	1.88	0.17	–	3869	1.38	0.24	198	5.87	0.01
**GGT**	4450	369.2	1×10^−4^	0.02	3952	138.0	1×10^−4^	0.04	333	23.90	1×10^−4^	0.08	3187	8.38	3×10^−3^	170	6.17	0.01
**BILIR**	5111	12.70	4×10^−4^	<0.01	4274	15.30	5×10^−3^	<0.01	333	0.76	0.39	–	3740	6.09	0.01	169	3.32	0.07
**PROT**	5086	11.65	7×10^−4^	<0.01	4271	0.96	0.1	–	334	5.29	0.03	–	3712	2.48	0.11	170	5.82	0.01
**GLOB**	5066	7.83	5×10^−3^	<0.01	4254	30.99	1×10^−4^	0.01	334	10.20	2×10^−3^	0.03	3700	9.53	2×10^−3^	170	4.06	0.04
**AST**	577	0.33	0.60	–	490	0.02	0.90	–	2	–	–	–	464	0.23	0.63	–	–	–
**ALP**	5120	104.9	1×10^−4^	0.03	4276	317.4	1×10^−4^	0.08	334	1.00	0.32	–	3744	47.94	1×10^−4^	170	0.45	0.50

Abbreviations: N = number of individuals, F = F test statistic, Chi^2^ = chi-square test, p = level of significance, R^2^ = Adjusted R^2^ explaining the proportion of the total variance explained.

¶ We report F-test statistics with 2 degrees of freedom from all regression analyses taking into account the relatedness between the twin pairs.

† For alcohol consumption truncated Gaussian regression was used due to the half normal distribution of the alcohol data.

BMI was significantly associated with ALT and GGT, in both the male and female twin cohort (p<10^−4^, R^2^ ranging from 2% for ALT in females to 11% for ALT in males), and ALB, BILIRB and ALP in female twin cohort only (p<10^−3^, R^2^ ranging from less than 1% for BILIRB to 8.2% for ALP).

We also checked for the effect of alcohol consumption on variation in the LFT proteins in males and females twin pairs respectively. Alcohol consumption showed suggestive association with the variation in ALB, GGT, TPROT and TGLOB in the male twin cohort (p<0.04). In the female twin cohort alcohol consumption was significantly associated with GGT, TGLOB and ALP (p<0.002).

### Path analysis and genetic model fitting of twin data

The results of genetic model fitting are shown in Table 4. For ALT, ALB, BILIRB, TPROT, TGLOB, AST and ALP, the ACE model – ascribing the total phenotypic variance to additive genetic, common and unique environments – was the best fitting model with narrow-sense heritabilities ranging from 15% in the case of TPROT to 48% in the case of ALP. For GGT the best fitting parsimonious model was AE model, with up to 77% of the total variation accounted for by additive genetic factors.

**Table 4:** Genetic model fitting results for variation in liver function test proteins.

Protein	X^2^	Df	ΔX^2^	ΔDf	P	Model	A (95% C.I.)	C (95% C.I.)	E (95% C.I.)
**ALT**	7.73	3	–	–	0.052	ACE	0.32 (0.19–0.44)	0.30 (0.21–0.34)	0.38 (0.34–0.44)
	44.44	4	36.71	1	0.000	AE	0.66 (0.62–0.70)	–	0.34(0.30–0.38)
	29.71	4	21.98	1	0.000	CE	–	0.50(0.46–0.53)	0.50(0.47–0.54)
**ALB**	6.38	3	–	–	0.095	ACE	0.20 (0.06–0.32)	0.36 (0.26–0.45)	0.44 (0.39–0.50)
	55.35	4	48.97	1	0.000	AE	0.62(0.58–0.66)	–	0.38(0.34–0.42)
	14.11	4	41.24	1	0.007	CE	–	0.48(0.44–0.52)	0.52(0.48–0.55)
**GGT**	8.53	3	–	–	0.036	ACE	0.69(0.58–0.78)	0.07(0.00–0.16)	0.24(0.21–0.27)
	10.90	4	2.37	1	0.124	AE	0.77(0.74–0.80)	–	0.23(0.20–0.26)
	128.03	4	119.50	1	0.000	CE	–	0.50(0.47–0.54)	0.50(0.46–0.53)
**BILIRB**	8.05	3	–	–	0.045	ACE	0.46 (0.37–0.56)	0.28 (0.20–0.36)	0.26 (0.23–0.29)
	45.83	4	37.78	1	0.000	AE	0.76(0.73–0.79)	–	0.24(0.21–0.27)
	81.81	4	73.75	1	0.000	CE	–	0.58(0.55–0.61)	0.42(0.39–0.45)
**TPROT**	23.18	3	–	–	0.000	ACE	0.15(0.00–0.26)	0.44(0.33–0.51)	0.41(0.35–0.46)
	100.69	4	77.51	1	0.000	AE	0.67(0.63–0.70)	–	0.33(0.30–0.37)
	30.22	4	7.04	1	0.008	CE	–	0.53(0.50–0.54)	0.47(0.44–0.50)
**TGLOB**	16.67	3	–	–	0.001	ACE	0.26 (0.17–0.35)	0.48 (0.48–0.54)	0.26 (0.23–0.30)
	148.28	4	131.60	1	0.000	AE	0.77(0.75–0.80)	–	0.23(0.20–0.25)
	45.61	4	28.94	1	0.000	CE	–	0.64(0.62–0.67)	0.36(0.33–0.38)
**AST**	4.82	3	–	–	0.185	ACE	0.40 (0.04–0.68)	0.34 (0.11–0.57)	0.26 (0.16–0.43)
	12.92	4	8.10	1	0.004	AE	0.78(0.69–0.85)	–	0.21(0.15–0.31)
	9.31	4	4.49	1	0.034	CE	–	0.57(0.48–0.65)	0.43(0.35–0.52)
**ALP**	1.37	3	–	–	0.714	ACE	0.48 (0.38–0.58)	0.24 (0.15–0.32)	0.28 (0.25–0.32)
	27.16	4	25.78	1	0.000	AE	0.74(0.71–0.77)	–	0.26(0.23–0.29)
	69.07	4	67.7	1	0.000	CE	–	0.55(0.51–0.58)	0.45(0.42–0.49)

The best fitting model is highlighted in grey. For each protein, full models (ACE & ADE) was compared to nested models (AE, CE, E) using a chi-squared test ΔX^2^ = (X^2^ sub model)−(X^2^ full model) with the degrees of freedom equal to ΔDf = (Df sub model)−(Df full model). The degrees of freedom increases from the full to sub or nested models due to drop in the numbers of parameters estimated as one moves down the model hierarchy. To be judged a good-fit, models should have a non-significant chi-squared goodness-of-fit statistic (p>0.05). Note, C and D cannot be included together in the same model as in quantitative genetic studies of human populations they are confounded thus the full model is either ACE or ADE. Comparisons with the ACE full model are shown here. In all cases, ACE provided a better model fit than ADE with a smaller chi-squared goodness-of-fit statistic (data not shown).

Abbreviations: X^2^ = chi-squared goodness-of-fit statistic; Df = degrees of freedom; ΔDf = (df sub model)−(df full model); Δ X^2^ = (X^2^ sub model)−(X^2^ full model); P = P-Value; A = Additive genetic influence; C = Shared environmental variance; E = Unique environmental variance.

## Discussion

In this study we used the classical twin design to gain insight into the relative contribution of genetic, environmental, as well as confounding effects of sex, age, body mass index and relative alcohol use to variation in the all major liver function test proteins.

Our results show that (1) in agreement with previous studies, variation in all the LFT proteins is highly variable; (2) a significant proportion of the variation (up to 11%) in the LFTs was due to confounding effects of age, sex, BMI, and alcohol use; (3) variation in all LFTs has a significant heritable basis; and (4) other than GGT, the LFTs are all affected to some extent by common environmental factors.

Our results are in general agreement with previous cited studies that have investigated the effects of age [22], [13], sex [10], [13], [23], BMI [13], [14], [22], and alcohol use [11], [12] on variation in the liver function test proteins. Age was shown to have a small but significant effect on all the LFT enzymes except ALB and AST.

Except total protein and total globulin, we observed significant sex differences with regards to variation in all the LFTs. For instance, our results show that up to 6% of the variation in bilirubin levels were due to sex. Furthermore, sex stratified analysis showed that BMI contributes significantly and differentially to the variation in many of the LFTs in our study. For example, up to 8% of the variation in ALP in females is due to variation in BMI whereas BMI contributes insignificantly to ALP variation in males. Moreover, although BMI is significantly associated to variation in ALT in both males and females the explained proportion of the variation in ALT by BMI is hugely different across sex groups; 2% in females and 11% in males. Finally, alcohol consumption was significantly associated with GGT, ALP, and total globulin in females. Whilst in the males, we showed that there was a trend of association with all the LFT proteins except ALT and ALP largely due to the fact that the male cohort was approximately 10 fold smaller than the female cohort. Interestingly, ALT, GGT and ALP were found to be significantly affected by all four confounding variable investigated - age, BMI, sex, alcohol use - highlighting the present dilemma and difficulty faced by clinicians in interpreting liver function test results.

Our study represents the largest and most extensive study to investigate the influences of genes and environment on the major LFT proteins. Our data shows that, quantitative variation in all the major LFT proteins – corrected for the effects of age, sex, BMI, and alcohol use – is under strong genetic control with narrow-sense heritability ranging from 15 to 77%. Minor differences were noted in the choice of the best fitting model for ALT, ALB, BILIRB and ALP, when we carried out sex stratified analyses, although point estimates of the A and C variance components were very similar (data not shown). We have also performed all our analysis by excluding male twins and our results were unchanged.

Our study is in general agreement with two previous studies. A small-scale study from the Danish twin registry consisting of 290 elderly twin pairs (age range between 73–102 years) were measured for a subset of the LFTs by Bathum et al [15]. In line with our results their study showed that variability in ALT, GGT, and BILIRB to be significantly heritable (35–61%). By contrast to our results, showed that additive genetic effects account for up to 20% of the variation in albumin levels, they showed no genetic effect for ALB.

A larger study by Whitfield et al. [24] based on twins from the Australian Twin Registry investigated the basis of variation and covariation in three of the LFT proteins including GGT, ALT and AST. In line with our results, Whitfield et al. showed that after correcting for the effects of sex and age, GGT, ALT and AST were highly heritable with additive genetic effects accounting for 52%, 48% and 32% of the variation in GGT, ALT and AST respectively. Moreover, half of the genetic variance in GGT was shown to be shared with ALT, AST, or both highlighting the significant role of pleiotropy in the case of variation in the LFTs. Although a thorough examination of the basis of covariation between all the LFT proteins studied is beyond the scope of this paper we did observe a high and significant phenotypic correlation between GGT & ALT (r^2^ = 0.5, p<1×10^−5^) as well as ALT and AST (r^2^ = 0.29, p<1×10^−5^) although GGT and AST levels were not correlated (r^2^ = −0.01, p = 0.78).

A major, previously unreported finding in our study is the strong influence of common environmental factors underpinning variation in the LFTs. Our result showed that variation in all but one of the LFT proteins (GGT) are strongly influenced by common environmental factors. In fact, in most cases the effect of common environment is either comparable or larger than the heritability of the trait. Although commonly highlighted as a strong motivation for undertaking a twin rather than a family based study, common environmental factors are seldom reported together with genetic factors in the same study. This is in large part due to lack of power in most twin studies to dissect the total familial component into its sub-components of genetic and common environments [25], [26]. Ignoring batch effects (our assays were designed to minimize batch effects), potential common environmental effects include stress, diet, and exposure to toxins, pathogens, radiation and chemicals. Although the relative contribution of these factors to variation in the LFTs would be purely speculative, one could argue that these factors along with other myriad factors could have a significant effect on an organ such as the liver which primarily functions as a site of metabolism, storage, decomposition, synthesis and detoxification. The observed strong influence of common environmental factors needs to be confirmed in a study with such environmental factors explicitly measured.

Although our study represents the largest and most extensive genetic epidemiological study of the LFT protein, there are a few potential limitations, which warrant further discussion. First, our study was conducted in twins, which have been previously shown to be representative of population-based samples for a wide range of common medical conditions and lifestyle characteristics [27]. Therefore, the conclusion derived from our study in twin population should be generalisable to the general population.

Second, although all the participant twins were deemed generally healthy – i.e. not suffering from any chronic diseases or taking prescribed medication - our results may have been influenced to an extent by unforeseen perturbations due to an infection, early stages of a disease, or a short course of an over the counter medication or dietary supplement [1]. Moreover, measurement errors, visit effects can all bias our results by inflating or deflating the within and between twin covariation in the LFT measurements even though care was taken to minimize the effects of these factors by using randomization of the samples prior to all biochemical analyses.

Third, due to non-technical reasons AST was only measured in a sub-sample of the female twin cohort (98MZ & 477DZ) and not in males at all. We are currently looking into increasing our sample size in the case of AST for future genetic association studies.

Fourth, due to historical reasons the TwinsUK database is predominantly female cohort and so we only had access to a limited number of male samples (no more than 500 twins).

Fifth, although not a major component of our study, the quality of data on alcohol consumption was not optimal. First, it was “recall” data collected from self-reported questionnaires and second our alcohol data is not representative of the drinking status of the individuals at the time at which the blood samples were taken for biochemical assays of the LFTs. For example, previously it has been reported that the amount of alcohol consumed in the past 30 days is an important indicator of high levels of GGT [23]. In our study, the time at which self-reported data on alcohol use was collected and the time at which blood samples were taken were up to 3 years apart. Both of these factors could reduce any association between alcohol consumption and the LFT proteins including GGT.

Finally, our conclusions are restricted to Northern European Caucasian populations and the results need to be replicated in other independent cohorts of different ancestry.

In conclusion, our study suggests the presence of sex differences and effect of age, BMI and alcohol consumption in variation of liver function proteins among individuals. Substantial heritability was observed for all the LFTs with the quantitative genetic modeling showing that a great proportion of the variation in liver function proteins are due to additive genetic effects stimulating further research to identify the responsible genes/region. Identifying the genes that influence the widely used liver function test will be very important in the future interpretation of their results and possibly reduce the need to further investigate subjects with high levels due to normal genetic variation.
